# The treatment efficacy of dupilumab in autosomal dominant hyper-immunoglobulin E syndrome with severe atopic dermatitis

**DOI:** 10.5415/apallergy.0000000000000121

**Published:** 2023-10-09

**Authors:** Weifeng Li, Qiqi Qi, Weipeng Wang, Dongqin Li

**Affiliations:** 1 Department of Dermatology, The First Affiliated Hospital of Zhengzhou University, Zhengzhou, Henan, China

**Keywords:** Dupilumab, eczema, hyper-IgE syndrome, primary immunodeficiency, STAT3

## Abstract

Hyper-immunoglobulin E syndrome (HIES) is a primary immunodeficiency disease characterized by atopic dermatitis, recurrent skin and lung infections, and significantly elevated serum immunoglobulin E levels. Autosomal dominant and loss-of-function pathogenic variants in the *STAT3* gene are the most common causes of the disease and studies have shown that the presence of IL-4 receptor (IL-4R) is upregulated in patients with dominant-negative mutations in the *STAT3* gene expression. Dupilumab is a monoclonal antibody that targets the IL-4α receptor and improves the symptoms of atopic dermatitis by inhibiting IL-4 and IL-13. We used dupilumab to treat severe dermatitis in a patient with STAT3-HIES and achieved satisfactory results.

## 1. Introduction

Hyper-immunoglobulin E syndrome (HIES) is a rare primary immunodeficiency disease that manifests as increased susceptibility to infection, eczema, and elevated serum immunoglobulin E (IgE) levels. There are 2 types of HIES: type I is autosomal dominant, the most common of which is the STAT3 pathogenic variant, and type II is autosomal recessive. Currently, there are no curative treatments for STAT3-HIES. Here, we report the successful treatment of severe dermatitis in a STAT3-HIES patient with a liver abscess and skin infection using dupilumab.

## 2. Case report

**Figure 1.**
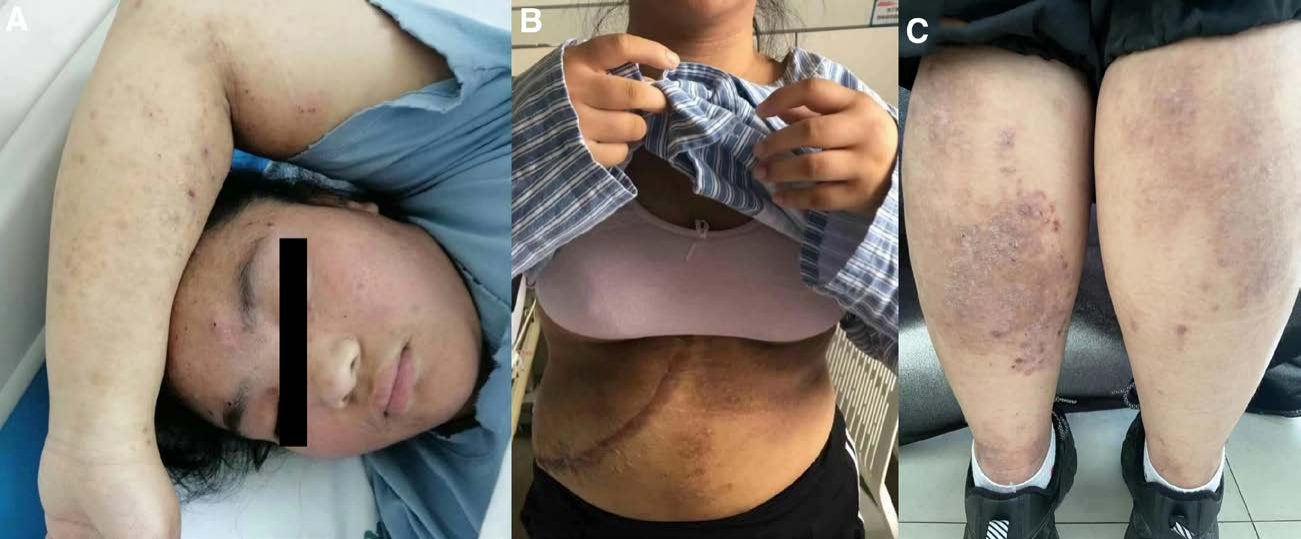
Skin manifestations before treatment with dupilumab. (A) Papules, nodules and pustules can be seen on the face. (B) Papules, large erythema and remaining pigmentation can be seen in the abdomen. (C) Inflammatory papules and erythema can be seen in both lower limbs, with dandruff on the surface and pigmentation after inflammation.

An 18-year-old female patient visited our hospital because of “erythema and papules all over the body with itching.” The patient’s skin was dry and rough throughout the body, and her eye distance and nosewing were wide. Diffuse dark brown pigmentation, scattered erythema, papules, and dander were observed on the face, trunk, and limbs, and nodules appeared on the hands with pain and swelling (Fig. 1). The absolute value of eosinophils was 570/µL (50–350/µL), and IgE>2,500.00 IU/mL (0–200 IU/mL). Imaging examination showed multiple liver abscesses and nodules in the abdominal wall and inflammation of the left lung. The patient’s test results from 3 years ago reflect that the absolute value of eosinophils was 820/µL (50–350/µL), IgE 14,789.00 IU/mL (0–200 IU/mL). Genetic tests showed that cytosine at position 1907 was mutated to thymine (c.1907C>T). According to the NIH-HIES, developed by the National Institutes of Health, the patient scored 45 points. Based on the medical history, the HIES diagnosis was caused by a STAT3 mutation. At the age of 6 months, the patient developed eczema-like changes all over her body. Since then, she has had intermittent multiple inflammatory nodules in the skin, abscesses with pain, and has been treated with “right hemi-hepatectomy” for a liver abscess, pneumonectomy for pulmonary hernias, and abdominal and peritoneal wall abscesses. Her blood cultures showed methicillin resistance with methicillin-resistant *Staphylococcus aureus*, and her parents and close relatives did not have a similar medical history.

**Figure 2.**
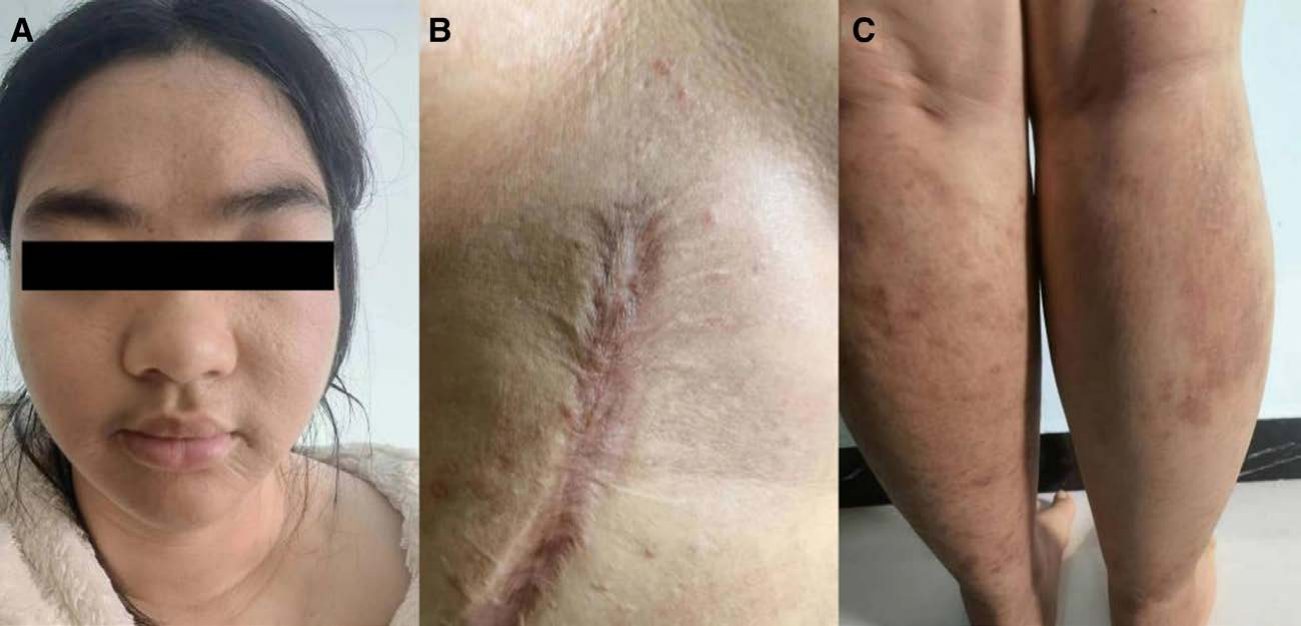
Dupilumab treatment of 28-week skin manifestation. (A) Complete resolution of facial lesions. (B) Complete resolution of abdominal lesions and postinflammatory hyperpigmentation. (C) Resolution of papules and erythema on both lower limbs, with residual postinflammatory hyperpigmentation.

The patient did not respond well to antihistamines (such as cetirizine/loratadine), topical glucocorticoids, tacrolimus ointment, or cephalosporins and sulfonamides. SCORAD (96/103) and DLQI scores (24/30) were calculated. After explaining the condition to the patient and her family, she was treated with dupilumab with a first subcutaneous injection of 600 mg, followed by subcutaneous injection of dupilumab 300 mg every 2 weeks. After 1 month of treatment, the patient’s skin manifestations and pruritus symptoms were significantly relieved. The patient has been regularly treated with dupilumab for 7 months with good tolerance. No obvious adverse reactions were observed during treatment. Erythema and papules on the face, limbs, and abdomen disappeared completely, leaving only a small amount of pigmentation (Fig. 2). The symptom of pruritus was significantly relieved, the scores for SCORAD and DLOI were (1/103) and (1/30), respectively. The reexamination results of relevant indicators showed that the absolute value of eosinophils was 720/µL (50–350/µL), and IgE was 4,220.00 IU/mL (0–200 IU/mL). Her liver and abdominal abscess did not relapse. The patient is currently undergoing treatment and follow-up.

## 3. Discussion

HIES comprises a heterogeneous group of patients with congenital immunodeficiency disorders. STAT3 autosomal dominant loss-of-function variants are the most common cause of HIES. Cytokines transduced by STAT3 include IL-6, IL-10, IL-11, IL-21, IL-22, and IL-23 [1]. Aberrant transduction of these pathways, coupled with the ubiquitous expression of STAT3, results in abnormal manifestations of the syndrome in multiple systems, including the skin, lungs, vessels, bone, and connective tissue. Currently, there is no cure for STAT3-HIES, and strengthening local skin care and preventive and therapeutic anti-*Staphylococcus* and antifungal agents remain the focus of treatment. Dupilumab treatment is recommended for eczema-like manifestations of STAT3-HIES [2]. In recent years, there have been reports on the use of dupilumab for the treatment of atopic dermatitis and other manifestations of HIES, such as asthma, allergic bronchopulmonary aspergillosis, and other atypical skin manifestations (including eosinophilic folliculitis and recurrent skin infections) [3, 4]. During dupilumab treatment, eosinophil levels increase in patients. We speculate that this may be related to the following factors: dupilumab blocks the role of IL-4 and IL-13 in eosinophil survival, activation, and recruitment to tissues, rather than excretion from the bone marrow, which is influenced by IL-5 [5]. Therefore, the use of dupilumab may result in a transient increase in blood eosinophils when initial treatment is administered.

Dupilumab is a monoclonal antibody against the IL-4α receptor that blocks the IL-4 receptor α chain (IL-4Rα) shared by the IL-4 and IL-13 receptors. IL-4 and IL-13 are key cytokines in developing atopic dermatitis, and expression of the IL-4Rα chain is upregulated in patients with a dominant-negative mutation in STAT3 [6]. We hypothesized that dupilumab inhibits IL-4 and IL-13 signaling by blocking IL-4Rα, resulting in decreased IL-4 and IL-13 cytokine-induced responses, including releasing pro-inflammatory cytokines, chemokines, and IgE. In addition, dupilumab downregulates many genes related to epidermal hyperplasia, dendritic cells, and T-cell activity, while upregulating genes are important for enhancing epithelial integrity [7]. After treatment, the liver abscess and skin infection symptoms were controlled and are still under observation. IL-4 inhibits Th1 and Th17 polarization [8] and reduces fibronectin expression in keratinocytes, thereby hindering wound healing [9]. Defects in the Th17 cytokines, IL-17, and IL-22, in patients with HIES offer possible molecular explanations for their selective susceptibility to skin and lung infections. Therefore, restoring IL-17 or IL-22 production represents a potential topical therapeutic approach for treating infections in patients with HIES [10]. Additionally, Th1 plays a role in controlling staphylococcal infections [11]. We speculate that controlling of liver abscesses and skin infections may be related to inhibiting IL-4 by dupilumab, thereby restoring the polarization of Th1 and Th17 cells and the production of IL-17 and IL-22. However, Matucci-Cerinic et al. [12] believed that dupilumab could restore Th1 polarization, whereas Th17 seemed unaffected.

## 4. Conclusion

Based on the current understanding of the pathogenesis of HIES, we conclude that dupilumab is effective for treating severe dermatitis in HIES. Further clinical, molecular, and genetic studies are needed to confirm this hypothesis regarding controlling liver abscesses and skin infections in patients treated with dupilumab.

## Acknowledgements

None.

## Conflicts of interest

The authors have no financial conflicts of interest.

## Authors contributions

Weifeng Li, Dongqin Li: Conception and design, acquisition of data, and drafting of the article. Weifeng Li, Weipeng Wang, Qiqi Qi: Formal analysis, investigation, and review and editing. All authors have read and agreed to the published version of the manuscript.

Written informed consent was obtained from the participant prior for the publication of this case report.
